# Low-level laser therapy for nipple trauma and pain during breastfeeding: systematic review and meta-analysis

**DOI:** 10.61622/rbgo/2025rbgo3

**Published:** 2025-03-17

**Authors:** Maria Victória Candida Gaitero, Ticiana Aparecida Alves de Mira, Edna Jéssica Lima Gondim, Simony Lira do Nascimento, Fernanda Garanhani Surita

**Affiliations:** 1 Universidade Estadual de Campinas Campinas SP Brazil Universidade Estadual de Campinas, Campinas, SP, Brazil.; 2 Universidade Federal do Ceará Fortaleza CE Brazil Universidade Federal do Ceará, Fortaleza, CE, Brazil.

**Keywords:** Low-level laser, Low level light therapy, Laser therapy, Breast feeding, Nipple trauma, Nipple pain, Nipples

## Abstract

**Objective:**

This study aimed to investigate the effect of low-level laser therapy (LLLT) on nipple trauma and pain during breastfeeding through a systematic review with a meta-analysis of selected studies.

**Source of the data:**

A thorough search was conducted on March 22, 2022, using the databases PubMed, SciELO, LILACS, PEDro, CINAHL, EMBASE, ScienceDirect, Scopus, Google Scholar, MEDLINE, the Cochrane Library, Clinical Trials, Web of Science, TRIP, DARE, and ProQuest. The search terms included various combinations of low-level laser therapy, nipple pain, nipple trauma, and breastfeeding.

**Studies selection:**

Out of 107 articles identified, only three controlled and randomized clinical trials was included. The extracted data encompassed breast and trauma characteristics, treatment types, outcomes (pain and healing process), evaluation tools, LLLT usage, laser brand, and parameters.

**Data collection:**

Data extraction was performed using RAYYAN for systematic reviews. The risk of bias in the studies was evaluated.

**Data synthesis:**

Pain was measured using the visual analog scale (VAS). The included studies did not use validated tools for assessing physical conditions. All studies employed LLLT with a 660-nm wavelength, though there were variations in equipment power, energy dose, and application methods. The meta-analysis revealed an average difference of −0.60 points (95% CI: −1.52 to 0.31) in the VAS pain scores between the LLLT and control groups. No heterogeneity was observed among the studies (I2=0%), indicating no significant difference in pain relief between LLLT (red light) and control groups.

**Conclusion:**

LLLT may offer a promising option for managing breastfeeding-related complications, though further research is required.

## Introduction

Breastfeeding represents the physiological bond of nourishment between a woman and her newborn, offering numerous benefits. It strengthens their affective connection and facilitates the transmission of immunological factors and enzymes, which have an important protective effect against common infections in childhood. Additionally, breastfeeding stimulates muscle tonus and the consequent development of bone, phonoarticular, and neurological structure, as well as reducing the risks of perinatal morbidity and mortality.^(1,2)^

Globally, only 44% of children are exclusively breastfed for the six months recommended by the World Health Organization. Pain resulting from nipple trauma is a cause of early weaning. This pain can restrict milk production and secretion, promoting maternal stress and inhibiting the production of oxytocin.^(2–4)^

Nipple trauma is defined as an injury to the areola and nipple integument, which can manifest as fissures, erosion, or ulceration of the skin, with clinical signs of erythema, edema, blisters, ecchymosis, and bleeding.^(5)^ Because nipple trauma significantly contributes to the interruption of exclusive breastfeeding, understanding effective management strategies is crucial.

Photobiomodulation (PBM), also called low-level laser therapy (LLLT), encompasses resources such as laser (light amplification by stimulated emission radiation) and light-emitting diodes. Non-thermal responses can promote analgesia and tissue repair, reducing the inflammatory process. The effect of laser therapy for pain relief was initially considered a secondary effect of dealing with the inflammatory state, but growing evidence exists that it can have a more direct effect on nerve conduction characteristics and hence directly result in reduced pain.^(6)^

The use of resources that reduce pain levels and improve healing in nipple trauma would be an advance in postpartum care. PBM has been shown to be an effective treatment in several areas of health, suggesting its potential efficacy in treating nipple trauma.^(7)^ However, the effectiveness of PBM and its parameters for use are not yet completely established in the literature. This review aimed to investigate the effect of LLLT on nipple trauma and pain during breastfeeding through a systematic literature search with a meta-analysis of selected studies.

## Methods

This is systematic review with meta-analysis designed according to the Preferred Reporting Items for Systematic Reviews and Meta-Analyses (PRISMA) checklist 2020.^(8)^

### Search strategy

We searched with no restriction of publication language or year. We developed and validated a search strategy, endorsed by the librarians responsible for the library of the Faculty of Medical Sciences at the University of Campinas. We searched for the following main MeSH terms: (low-level laser therapy OR low-level light therapy OR laser therapy OR low-level laser therapy OR LLLT OR low-level laser OR low-level) AND (nipple pain OR nipple trauma) AND (breastfeeding) using the following databases: PubMed, SciELO, LILACS, PEDro, CINAHL, EMBASE, ScienceDirect, Scopus, Google Scholar, MEDLINE, the Cochrane Library, Clinical Trials, Web of Science, TRIP, DARE, and ProQuest.

### Inclusion criteria

Concerning the inclusion criteria, we searched for all randomized clinical trials, quasi-experimental studies, non-randomized clinical trials, case-control studies, cohort studies, and transversal studies that reported the use of LLLT in women with nipple trauma or pain during breastfeeding.

### Selection strategy

We conducted a thorough search on March 22, 2022, which was the last time we found new data. We also conducted a simple search in December 2023, finding no new published studies. Two researchers (MVCG, TAAM) extracted the data using the RAYYAN—Intelligent Systematic Review tool in two phases.^(9)^ The primary screening was based on the title and abstract. After excluding irrelevant articles, we read each study in its entirety to determine its eligibility. For any discrepancies between reviewers, a third opinion (FGS) was requested (Supplementary Material 1).

### Data extraction

A standard form was used for the data extraction. The data extracted included the characteristics of the breasts and trauma (nipple type; skin, nipple, and areola color), types of trauma treatment, outcomes (pain and healing process, satisfaction), evaluation tools, and evaluation time (when and how many times the pain and the healing process were evaluated). We also included data such as population characteristics, obstetric characteristics, study design, publication year, and sample size. Regarding the usage of LLLT, we included the laser brand, wavelength, and parameters (power, energy density, irradiance); the number of applications; and adverse effects of the applications.

### Evaluation of outcomes

The primary effect measurements were the differences between the intervention and control groups in the level of pain during breastfeeding and the healing process of the nipple trauma. Additional secondary outcomes such as satisfaction and adverse effects were included.

### Bias analysis

The risk of bias was evaluated independently by two authors (MVCG, TAAM) using the Cochrane risk-of-bias tool for randomized trials (RoB), a standard tool from the program Review Manager 5.4. The RoB tool assesses random sequence generation (selection bias), allocation concealment (selection bias), blinding of participants and personnel (performance bias), blinding of outcome assessors (detection bias), incomplete outcome data (attrition bias), selecting reporting (reporting bias), and other bias.^(10)^ We also used the Grading of Recommendations, Assessment, Development and Evaluations (GRADE) for the classification of the evidence quality and purpose recommendations for clinical practice. The GRADE approach suggests the initial classification of randomized controlled trials as high-quality studies (score 4), with a reduction to moderate, low, or very low depending on [1] the quality of the original studies, [2] inconsistency of the results (heterogeneity), [3] indirect evidence, [4] imprecision, and [5] publication bias.

### Data synthesis

The data extraction was performed by two independent reviewers (MVCG, TAAM) using a form tailored to the requirements of this systematic review. Any disagreement or discrepancy between the reviewers was resolved through discussion with a third member of the review team (SLN). The data extracted from each study for the meta-analysis included the number of participants per group, the mean pain intensity before and after LLLT irradiation for each group, and the standard deviation for each mean. Means and a measure of dispersion (standard deviation [SD], standard error [SE], or 95% confidence interval [CI]) were extracted for each group. SE was converted to SD by the equation SD= SE × (n) if the SD was not provided in the original study. If a study used more than one irradiation, only data from the first was considered. A single intervention and control group was used for each study in the meta-analysis. The meta-analysis combined the results of all the included papers and examined the effect of LLLT interventions on pain during the postpartum period compared to placebo irradiation as a control group. When necessary, the authors were contacted for data recovery. Studies not providing data on the baseline or end-point outcomes were excluded from the meta-analysis. The outcome of interest for the analyses was the mean VAS pain change from before to after LLLT irradiation, with a 95% CI. A random-effect model and inverse variance method were used. The heterogeneity was assessed using the Cochran Q test, which considers the deviations of each study effect size in relation to the general estimate. The variation between studies was quantified using I² statistics (considered large if ≥50%). A funnel plot was generated, and the Egger regression test was performed to assess publication bias. All analyses were performed using Review Manager (RevMan) version 5.4.

## Results

### Study selection and characterization

**Figure 1:**
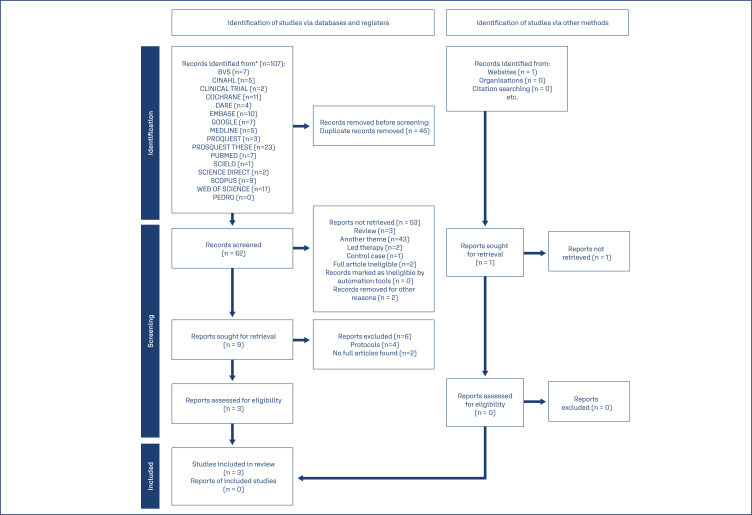
Preferred Reporting Items for Systematic Reviews and Meta-Analyses (PRISMA) flowchart of the study screening process.

Of 107 articles found, 45 were excluded as duplicates. After the analysis of the titles and abstracts, we excluded 53 articles. Finally, nine articles were selected for full-text evaluation, and six were excluded: four articles were protocols, and two were not fully available (one because it was a congress poster and another due to a lack of availability, even after contact with the authors).^(11)^ The PRISMA flowchart shows the selection steps and reasons for article exclusion (Figure 1).^(8)^

Thus, three controlled and randomized clinical trials were included in this study.^(12–14)^ Two of these were blinded, and all were conducted in Brazil. The details of the studies included are described in chart 1.

**Chart 1:** Features of trial design, participants, evaluation tools, intervention, and outcomes of the included studies

Author/ year/ country	Study design	Population	Sample size n(%)	Outcomes	Evaluation time	Evaluation tool	Device brand and parameters	Intervention time	Number of applications	CG	Outcome: Pain	Outcome: Healing process	Outcome: Satisfaction	Side effects
Curan et al. (2023)^(12)^ Brazil	RCT	IC: NT, pain EC: history of breast malignant disease, breast implants, use of medication, mastitis, cognitive deficit, PTS .	Total=54(100) CG=16(29,62) IG=20(37,03) Other=18(33,33)	– Pain – Length of lesion – before and after the last laser application.	3 moments before the application and 3 moments after the application (before, during, and after BF)	– VAS – Measuring tape to evaluate the extension of the lesion	Laser DMC EC - Spectrum Red (660nm) Power: 100mW Power density: - Energy released: 1J e 3J	3 days in a row after the intervention (every 24 hours).	1J on the centre of the lesion, and 3J on 8 points around the areola	Others: laser ILIB for 30 minutes CG: Information about breast care	Reduction in IG In the IG (*p*=.002) – after the laser application	Decrease in the lesion size in the IG 1 (*p*=.042), and the IG 2 (*p*=.006)	Data not collected	No side effects
Coca et al. (2016)^(13)^ Brazil	RCT, blinded	IC: NT, BW > 2500 g, exclusive BF EC: TSU, inverted or pseudo-inverted nipples; mastitis, malignant lesions, PTS	Total=59(100) CG=29(49,15) IG =30(50,84)	– Breasts conditions – Nipple colour – Pain	1^st^ contact, 24 and 48 hours after the first application	VAS	Laser MMO Spectrum Red (660nm) Power: 40mW Energy density: 5w/cm² Energy per point: 0,2J	After the 1ª evaluation, after 24 and 48 hours.	Irradiation on 3 consecutive points distributed over the lesion – centre, left and right extremities	Placebo irradiation, with a red-light-emitting diode with sound, but with no therapeutic effect	Reduction in IG Before 1^st^ irradiation (*p*=.05) (decrease 1.3cm in VAS) after 2^nd^ irradiation *p*=.016 (decrease of 2cm in VAS)	Data not collected	Data not collected	Data not collected
Camargo et al. (2020)^(14)^ Brazil l	RCT, blinded	IC: NT, BW> 2500 g, exclusive BF EC: twins, inverted or pseudo-inverted nipples, mastitis or malignant disease, TSU, PTS, discharge before 24 h.	Total=80(100) CG=40(50) IG= 40(50)	– Breasts conditions – Nipple and breast conditions – Nipple colour – Position and lesion in each nipple – Pain – Side effects	Before the laser application, 6 and 24 hours after application	– Measuring tape to evaluate the extension of the lesion – VAS – Form about the side effects	Laser MMO Spectrum Red (660nm) Power: 100mW Power density: 3.3w/cm² Energy released: 2J Energy density: 66.6J/cm² Time application: 20 seconds	Single application after the evaluation	20 seconds of irradiation on the centre of the lesion	Placebo irradiation, with a red-light-emitting diode with sound, but with no therapeutic effect (blocked with aluminium foil inside the equipment)	Reduction in IG *p*=0.419	No comparison before and after intervention	80% of participants (IG) were satisfied with the procedure, reporting relief in pain.	Most report in IG: tingling (*p*=.032)

BW: birth weight; BF: breastfeeding; CG: control group; EC: exclusion criteria; IC: inclusion criteria; IG: intervention group; ILIB: Irradiation Laser Intravascular of Blood; J: joule; J/cm²: joule per centimetre²; mW: milliwatt; NM: nanometres; NT: nipple trauma; PTS: photosensitivity; RCT: randomised clinical trial; TSE: topical substance use; VAS: visual analogue scale; W/cm²: watt per centimetre². In all studies, the hospital staff oriented all the participants about breastfeeding

### Sociodemographic and clinical characteristics

In all of the articles, the total sample was 193(100%) participants with nipple trauma or pain during breastfeeding, aged between 18 and 40 years. Concerning the obstetric characteristics in general, 96(49,74%) participants were multiparous, 97(50,25%) were primiparous, 73(37,82%) had cesarian births, 120(62,17%) had vaginal births, and the gestational age ranged from 36–39 weeks.^(12,14)^ A total of 197 breasts had nipple trauma.^(12,14)^ Only Curan et al.^(12)^ characterized the nipple trauma, describing excoriation (n=63), fissure (n=27), and erosion (n=11) among the 101 traumas included. Camargo et al.^(14)^ reported on which side the nipple trauma occurred (right: n=45; left: n=51) but did not characterize the traumas. Coca et al.^(13)^ did not describe the nipple traumas in any way. In Curan et al.,^(12)^ 48 women presented non-protruding nipples and six presented protruding ones, whereas in the study of Camargo et al.,^(14)^ the majority of women (n=60) presented protruding nipples.^(12,14)^ Regarding the nipple color, most of the women (n=130) presented brown nipples.^(13,14)^

### Measurement process

Pain was measured with the VAS in all three studies. The timing of the pain measurement varied between studies. Before the intervention, the three studies assessed pain: one study at the first contact,^(13)^ another before the laser application,^(14)^ and another before, during, and after breastfeeding.^(12)^ After the intervention, assessments were conducted at 24 and 48 hours, with a new laser application performed within this interval,^(13)^ evaluations immediately after application, at 6 hours and 24 hours,^(14)^ and before, during, and after breastfeeding.^(12)^ The studies included did not use validated tools to evaluate the physical condition of the breasts, nipples, or nipple trauma. Curan et al.^(12)^ and Camargo et al.^(14)^ used a technique to measure the healing process (respectively, measuring the length and width before and after treatment, and using a measuring tape to assess the extension of the trauma). Coca et al.^(13)^ did not include any measurements in the data.

### Intervention

All studies used LLLT with a 660-nm wavelength (red spectrum). Some variations existed in the power of the equipment: 40 mW^(13)^ and 100 mW.^(12,14)^ The energy dose used and the application method also varied: 0.2 J of power at three points of trauma (center, left, and right) on three consecutive days;^(13)^ a single irradiation of 2 J in the center of the trauma;^(14)^ and a single irradiation in the center of the trauma with 1 J and eight points with 3 J around the areola.^(12)^ All studies used only red laser as the intervention. Infrared irradiation and other approaches to treat nipple trauma were not used in any of the studies. As part of hospital protocol, all the women received an orientation to breastfeeding,^(12–14)^ which consisted of information regarding the proper position to breastfeed, how to make the baby latch, and how to take care of the breasts.^(13,14)^ In one study, the control group was also provided with an orientation to proper breastfeeding: position (mother and newborn), breast massage and milk pumping, latching, newborn sucking patterns, and use of accessories (relief donuts).^(12)^ The intervention was blinded in two studies. One used a non-therapeutic red light to simulate the application of irradiation in the control group.^(13)^ Another used a non-therapeutic red light and a barrier of aluminum foil inside the equipment, both to simulate the time and sound of the application.^(14)^ In the non-blinded study, besides the control group (which did not receive the laser), a third group received a different intervention, the intravascular laser irradiation of blood (ILIB) technique.^(12)^

### Summary of outcomes

#### Pain

**Figure 2:**
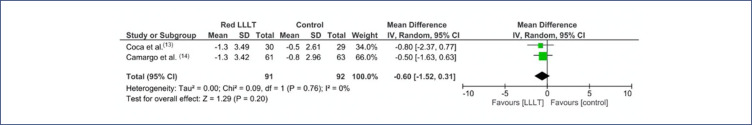
Forest plot of two studies: relief of pain after the use of low-level laser in nipple trauma, outcome: nipple pain

Coca et al.^(13)^ described a reduction in pain levels in the intervention group after the first (p=.050) and second (p=.016) laser applications. No significant reduction in pain (p=.419) was found by Camargo et al.^(14)^ Curan et al.^(12)^ reported a decrease in the pain level before breastfeeding in the groups that received the laser and ILIB applications compared to the control group (p=.002). Data from two of the three studies were included in the meta-analysis because Curan et al.^(12)^ did not present VAS data for the control group after the irradiation, only for the ILIB comparison group. The meta-analysis (Figure 2) showed an average difference of −0.60 points (95% CI: −1.52 to 0.31) in the pain VAS between the intervention (LLLT) and control groups. No heterogeneity existed among the studies (I^2^=0%), and no difference in pain relief was found between the application of LLLT (red light) and control groups.

We observed a very low evidence quality, with severe risk of bias, inconsistency, indirectness, and imprecision, as assessed through GRADE analysis. The absolute effect with a 95% confidence interval showed mean difference 0.6 lower (1.52 lower to 0.31 higher). The symmetry displayed in the funnel plot suggests an absence of publication bias.

#### Healing process

For the groups that received LLLT (p=.042) and the one that received ILIB (p=.006), Curan et al.^(12)^ found a decrease in the area size of the lesions. Camargo et al.^(14)^ did not describe any results regarding the healing process, and Coca et al.^(13)^ did not evaluate this parameter.

#### Adverse effects and satisfaction

Camargo et al.^(14)^ identified that a tingling sensation was more frequent in the patients who received the laser treatment compared to controls (p=.032). Of all the women in that study, 80% reported that "the laser helped a lot" and "relieved the pain during breastfeeding." Curan et al.^(12)^ did not report any adverse effects and did not evaluate the women's satisfaction. Coca et al.^(13)^ evaluated neither of these outcomes.

#### Bias risk

**Figure 3:**
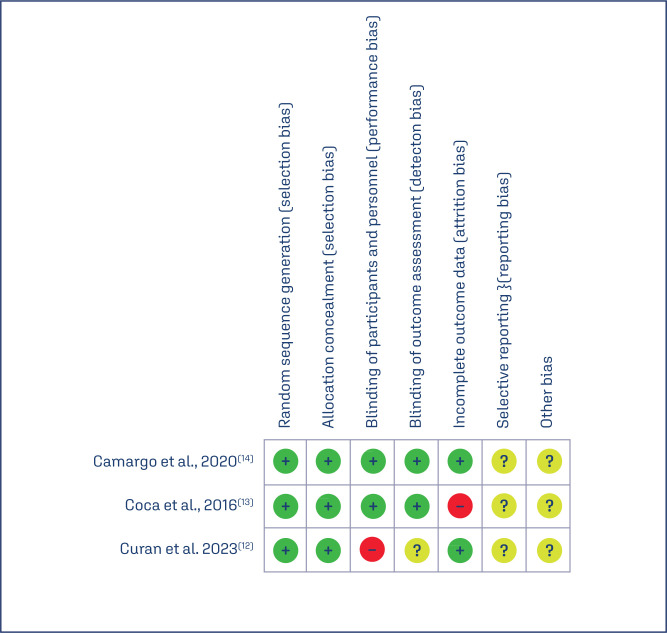
Risk of bias summary: review the authors’ judgments about each risk of bias, based on the Cochrane risk-of-bias assessment tool. Green colour indicates low risk of bias, yellow colour means unclear risk of bias, and red colour indicates high risk of bias

Figure 3 presents the risk of bias in the three included studies. We identified a high bias risk for blinding in the study of Curan et al.,^(12)^ and the other studies did not include complete data.^(12–14)^ Regarding the other criteria, the majority presented a low or uncertain risk of bias for the selective bias and other biases.

## Discussion

This systematic review reveals the lack of studies on the effects of laser on breastfeeding. It found very few published articles evaluating the use of LLLT in treating nipple pain and trauma, among the main complications of breastfeeding. The number of participants evaluated was small, and the parameters that were used among the studies varied. Despite these points compromising the conclusions, the analysis shows an inclination toward positive effects for these outcomes when we examine the results individually. However, this meta-analysis did not demonstrate a significant difference in the pain associated with nipple trauma in women during the postpartum period, and it showed a lack of data to prove the effect of laser in the healing process.

The use of infrared lasers is well-known for promoting analgesia due to its effect on a cellular level, promoting an increase in endogenous opioid neurotransmission in local blood flow and deceleration or blockage of the conduction of central and peripheral nerve fibers, leading to a reduction in pain levels.^(15)^ In that respect, none of the studies included in this review used the specific wavelength responsible for the analgesic effect suggested in the literature (830–904 nm). This corroborates our findings about the significant difference in the pain level of the meta-analysis outcomes.

The presence of tissue trauma might be related to pain. Understanding the healing process and promoting its acceleration are thus important. The literature suggests the use of red laser (with a wavelength between 630 and 690 nm) for healing superficial wounds since the light radiation acts in the superficial layer of the dermis.^(16)^ The laser reduces inflammation, promoting an increase in the constitution of granulation tissues and the proliferation of fibroblasts, synthesis of components of extracellular matrix (especially collagen), neovascularization, and epithelialization.^(17)^

After 72 hours of LLLT (wavelength 660 nm and energy 5 J/cm²), histological analysis showed increases in proteic synthesis and vascular endothelial growth factor, which corroborates the speeding up of the healing process.^(18)^ Therefore, analyzing the outcomes shows that calibrating the LLLT equipment parameters, such as wavelength, power, energy density, and energy per point, is crucial to suit the demand of each treatment. However, the literature still lacks studies using LLLT and its parameters for each practice purpose.

Regarding the usage of LLLT in other tissues, two studies were found that evaluated the effect of LLLT for promoting relief in cesarian section. One used wavelengths of 804 and 650 nm, with power energy of 1 J of red light and 2 J of infrared light over the sutured section.^(19)^ The other used a wavelength of 604 nm, and two protocols were followed: one with 0.12 J of power energy for four seconds, and one with 0.24 J for eight seconds, on 10 points along the section, totaling 2 J/cm² and 4 J/cm².^(7)^ Both studies found significant results for pain relief in cesarean section after the use of LLLT. Despite the positive findings of these studies, some contrary results have been found for other tissues. No positive results were found after episiotomy, as demonstrated by Alvarenga et al.^(17)^ and Santos et al.^(20)^ Neither of these studies used similar LLLT parameters. One used a wavelength of 780 nm and 0.2 J energy per point, for 10 seconds, totalizing 5J/cm².^(17)^ The other used a wavelength of 660 nm and 0.15 J energy per point for 10 seconds, totaling 3.8 J/cm².^(20)^

Currently, research is wide on the use of LLLT in musculoskeletal pain. A meta-analysis of 18 studies, evaluating the effect of laser on joint pain, identified that LLLT brought pain relief for joints with dysfunctions, such as the knee, shoulder, and back, demonstrating an analgesic effect.^(21)^ It could also reach deeper tissues, despite the various parameters adopted by the studies.

LLLT is a common resource in clinical practice that is applied for the treatment of pain and acceleration of the healing process in several tissues. However, the scientific literature still presents some gaps in the results and possible positive effects of this technique. Confirming the effects of the laser and treatment outcomes is hindered by some points, such as the diversity of the tissues treated by the laser, the parameters adopted for each clinical condition, the technique applied during irradiation, and the evaluation methods.

None of the clinical trials included in this review described any validated instrument for evaluating nipple trauma resulting from breastfeeding or the evolution of healing. Only one study described the results of the healing process, using a measuring tape assessment.^(14)^ A review described 18 methods for evaluating nipple trauma, such as scales, indexes, or scores; lesion measurement; clinical examination; the use of magnifying glasses and enlarged photographs; and telephone calls.^(22)^ Recently, the same group of researchers developed a validated instrument for the classification of nipple and areolar lesions resulting from breastfeeding, which contributes to the evaluation of nipple trauma.^(5,22)^ However, there is a lack of validated data to assess the evolution of the healing process and, consequently, to evaluate the effect of laser treatment on injuries to the nipple tissue. This could be a field of research for new studies in the area.

As deficient points, we highlight the scarcity of published studies on this subject and the high heterogeneity in the LLLT parameters used for the treatment of nipple trauma (pain and healing). Additionally, the results of this meta-analysis consider only one single laser irradiation, which is not a reality in clinical practice. Thus, the results could not show the full effect of LLLT since nipple pain and trauma are the result of not only lesion tissue but also external factors such as the breastfeeding position and newborn sucking. As a positive point, this is the first review that analyzes the topic and assesses the main outcomes of pain and analgesia related to nipple injuries. This study can be used as a guide for others in the area.

The findings of this study suggest an open field regarding the use of laser for nipple trauma, with important gaps to be filled. New studies could consider a larger sample size, the use of distinct laser parameters (wavelength, power, energy, energy per point, density of energy, time of irradiation, and method of irradiation) to compare and validate the method, and other evaluation methods for the validation of the healing process. These new data might positively impact the main evidence expected in this population: pain relief and accelerated healing to maintain exclusive breastfeeding as recommended by the World Health Organization.^(4)^

## Conclusion

This systematic review of the effects of LLLT revealed no difference in pain associated with nipple trauma in puerperal women, as well as a lack of data to prove the effects of laser on the healing of these lesions. The review also highlights the scarcity of standardized methods for evaluating the healing process of nipple and areolar tissues. The limited number of studies on the use of lasers in breastfeeding complications, the small number of participants included, and the divergences in the parameters used emphasize the need for more comprehensive and methodologically consistent research. The current inclination towards positive effects of LLLT for the outcomes evaluated in this study suggests that it may be a promising therapeutic option in managing complications associated with breastfeeding.

## Supplementary Material 1. Low-level laser therapy for nipple trauma and pain during breastfeeding: systematic review and meta-analysis

**Supplementary Material**

**Search Strategy**

**Descriptors and terms:**

**Table:**

Descritor em português:	Terapia com Luz de Baixa Intensidade
Descritor em inglês:	Low-Level Light Therapy
Descritor em espanhol:	Terapia por Luz de Baja Intensidad
	Espanhol da Espanha
Descritor em francês:	Photothérapie de faible intensité
Termo(s) alternativo(s):	Bioestimulação a Laser
	Irradiação a Laser de Baixa Intensidade
	Irradiação a Laser de Baixa Potência
	LLLT
	Terapia a Laser de Baixa Intensidade
	Terapia a Laser de Baixa Potência
Código(s) hierárquico(s):	E02.594.540
	E02.774.500
	MT3.685.735
Identificador Único RDF:	https://id.nlm.nih.gov/mesh/D028022
Nota de escopo:	Tratamento que usa radiação com luz de baixa intensidade de forma que os efeitos são uma resposta à luz e não ao calor. Várias fontes de luz, especialmente os lasers de baixa intensidade são usados.

**Table:**

MESH	"Low-Level Light Therapy" OR "Light Therapies, Low-Level" OR "Light Therapy, Low-Level" OR "Low Level Light Therapy" OR "Low-Level Light Therapies" OR "Therapies, Low-Level Light" OR "Therapy, Low-Level Light" OR "Photobiomodulation Therapy" OR "Photobiomodulation Therapies" OR "Therapies, Photobiomodulation" OR "Therapy, Photobiomodulation" OR LLLT OR "Laser Therapy, Low-Level" OR "Laser Therapies, Low-Level" OR "Laser Therapy, Low Level" OR "Low-Level Laser Therapies" OR "Laser Irradiation, Low-Power" OR "Irradiation, Low-Power Laser" OR "Laser Irradiation, Low Power" OR "Low-Power Laser Therapy" OR "Low Power Laser Therapy" OR "Laser Therapy, Low-Power" OR "Laser Therapies, Low-Power" OR "Laser Therapy, Low Power" OR "Low-Power Laser Therapies" OR "Low-Level Laser Therapy" OR "Low Level Laser Therapy" OR "Low-Power Laser Irradiation" OR "Low Power Laser Irradiation" OR "Laser Biostimulation" OR "Biostimulation, Laser" OR "Laser Phototherapy" OR "Phototherapy, Laser"

OR

**Table:**

TERMO LIVRE	"Low-level laser therapy (LLLT) "OR "Laser Therapy" OR "Low Level Laser Therapy (LLLT) " OR LLLT OR "low-level laser" OR "photobiomodulation therapy" OR "low-level laser therapy" OR "Low-Level"

AND

**Table:**

Descritor em português:	Ferimentos e Lesões
Descritor em inglês:	Wounds and Injuries
Descritor em espanhol:	Heridas y Lesiones
	Espanhol da Espanha
Descritor em francês:	Plaies et blessures
Termo(s) alternativo(s):	Ferida
	Feridas
	Ferimento
	Ferimentos
	Ferimentos e Traumatismos
	Lesão
	Lesões
	Trauma
	Traumas
	Traumatismo
	Traumatismos
Código(s) hierárquico(s):	C26
Identificador Único RDF:	https://id.nlm.nih.gov/mesh/D014947
Nota de escopo:	Danos infligidos no corpo como resultado direto ou indireto de uma força externa, com ou sem rompimento da continuidade estrutural.

**Table:**

MESH	"Wounds and Injuries" OR "Injuries and Wounds" OR "Wounds and Injury" OR "Injury and Wounds" OR "Wounds, Injury" OR Trauma OR Traumas OR "Injuries, Wounds" OR "Research-Related Injuries" OR "Injuries, Research-Related" OR "Injury, Research-Related" OR "Research Related Injuries" OR "Research-Related Injury" OR Injuries OR Injury OR Wounds OR Wound

OR

**Table:**

Descritor em português:	Cicatrização
Descritor em inglês:	Wound Healing
Descritor em espanhol:	Cicatrización de Heridas
	Espanhol da Espanha
Descritor em francês:	Cicatrisation de plaie
Termo(s) alternativo(s):	Cicatrização de Feridas
	Cicatrização de Ferimentos
Código(s) hierárquico(s):	G16.762.891
Identificador Único RDF:	https://id.nlm.nih.gov/mesh/D014945
Nota de escopo:	Restauração da integridade a tecido traumatizado.

**Table:**

MESH	"Wound Healing" OR "Healing, Wound" OR "Healings, Wound" OR "Wound Healings"

OR

**Table:**

TERMO EMTREE	"tissue repair" OR "repair, tissue"

OR

**Table:**

Descritor em português:	Dor
Descritor em inglês:	Pain
Descritor em espanhol:	Dolor
	Espanhol da Espanha
Descritor em francês:	Douleur
Termo(s) alternativo(s):	Sofrimento Físico
Código(s) hierárquico(s):	C23.888.592.612
	F02.830.816.444
	G11.561.790.444
Identificador Único RDF:	https://id.nlm.nih.gov/mesh/D010146
Nota de escopo:	Sensação desagradável induzida por estímulos nocivos que são detectados por TERMINAÇÕES NERVOSAS de NOCICEPTORES.

**Table:**

MESH	Pain OR "Pain, Burning" OR "Burning Pain" OR "Burning Pains" OR "Pains, Burning" OR "Suffering, Physical" OR "Physical Suffering" OR "Physical Sufferings" OR "Sufferings, Physical" OR "Pain, Migratory" OR "Migratory Pain" OR "Migratory Pains" OR "Pains, Migratory" OR "Pain, Radiating" OR "Pains, Radiating" OR "Radiating Pain" OR "Radiating Pains" OR "Pain, Splitting" OR "Pains, Splitting" OR "Splitting Pain" OR "Splitting Pains" OR Ache OR Aches "Pain, Crushing" OR "Crushing Pain" OR "Crushing Pains" OR "Pains, Crushing"

AND

**Table:**

Descritor em português:	Mamilos
Descritor em inglês:	Nipples
Descritor em espanhol:	Pezones
	Espanhol da Espanha
Descritor em francês:	Mamelons
Código(s) hierárquico(s):	A01.236.500
Identificador Único RDF:	https://id.nlm.nih.gov/mesh/D009558
Nota de escopo:	Órgãos cônicos os quais usualmente fornecem passagem ao leite proveniente das glândulas mamárias.

**Table:**

MESH	Nipples OR Nipple OR Areola OR Areolae

OR

**Table:**

TERMO LIVRE	"nipple pain" or "nipple trauma" OR "nipple fissures"

AND

**Table:**

Descritor em português:	Aleitamento Materno
Descritor em inglês:	Breast Feeding
Descritor em espanhol:	Lactancia Materna Espanhol da Espanha
Descritor em francês:	Allaitement naturel
Termo(s) alternativo(s):	Aleitamento
	Aleitamento Materno Exclusivo
	Alimentado ao Peito
	Alimentado no Peito
	Alimentação ao Peito
	Amamentado
	Amamentação
	Amamentação com Ama-de-Leite
	Compartilhamento de Leite
Código(s) hierárquico(s):	F01.145.407.199
	G07.203.650.195
	G07.203.650.220.500.500
	G07.203.650.353.199
	SP6.180.632.301.236
	SP6.810.304.526.225
Identificador Único RDF:	https://id.nlm.nih.gov/mesh/D001942
Nota de escopo:	Amamentação de um lactente no peito da mãe.

**Table:**

MESH	"Breast Feeding" OR Breastfed OR Breastfeeding OR "Breast Fed" OR "Milk Sharing" OR "Sharing, Milk" OR "Breast Feeding, Exclusive" OR "Exclusive Breast Feeding" OR "Breastfeeding, Exclusive" OR "Exclusive Breastfeeding" OR "Wet Nursing"
